# An unusual occurrence of penetrating aortic arch injury by a ball-point pen: a case report and review of the literature

**DOI:** 10.1186/s13019-022-02057-5

**Published:** 2022-12-15

**Authors:** Herbert Ariaka, John Paul Magala, Naomi Kebba, Ronald Kabuye, Stella Magara Namirembe, Tom Philip Mwambu, Kenneth Ahabwe, Miriam Nalule

**Affiliations:** 1 Department of Cardiovascular & Thoracic Surgery, Uganda Heart Institute, P.O BOX 7051, Kampala, Uganda; 2 Department of Thoracic & Vascular Surgery, Mulago National Referral Hospital, P.O Box 7051, Kampala, Uganda; 3 Department of Anaesthesia, College of Health Sciences, Makerere University, P.O Box 7060, Kampala, Uganda

**Keywords:** Penetrating aortic arch injury, Ball-point pen, Primary aortic arch repair

## Abstract

**Background:**

Aortic arch injuries account for about 8% of thoracic aortic injuries. Penetrating zone I neck injuries account for 18% of vascular injuries in the neck and have great potential to traverse to involve thoracic vascular structures as well. The hard and soft signs of vascular injury facilitate triage of patients on an individual basis. We present a case of a ball-point pen traversing through zone I of the neck and causing penetrating aortic arch injury with minimal mediastinal haemorrhage.

**Case presentation:**

We present a polytrauma patient who was admitted with traumatic brain injury and a ball-point pen lodged above the sternal notch in zone I of the neck following a road traffic accident. He underwent mediastinal exploration via a median sternotomy. The ball-point pen was found penetrating the anterior wall of the aortic arch and resting in its lumen. The ball-point pen was successfully explanted and primary repair of the penetrating aortic arch injury was done. He had an uneventful recovery without any added secondary neurological complications.

**Conclusion:**

Penetrating aortic arch injuries are rare compared to injuries of the ascending aorta and descending aorta. They require a high index of suspicion, rapid investigation and urgent intervention in view of their high associated fatality. The ball-point pen in this case assumed the shape of a plug which acted as a seal at the site of injury preventing catastrophic exsanguination.

## Background

Aortic arch injuries account for about 8% of thoracic aortic injuries. The reported mechanisms of penetrating thoracic vascular injuries include gunshots, stab wounds, and iatrogenic injuries [1, 2]. Penetrating injuries to the thoracic aorta carry a higher mortality compared to injuries occurring at other parts of the aorta. One study found that patients with penetrating abdominal aortic injury were three times more likely to survive than those with thoracic aortic injuries [3]. This case report presents a rare case of penetrating aortic arch injury from a ball-point pen with access via the suprasternal area. The proximity of the lodged pen to a major vessel anatomical area and history of torrential bleeding at the site of the accident were the soft signs of vascular injury in this patient, who was otherwise hemodynamically stable.

## Case presentation

A 20-year-old male was admitted to our emergency department as a referral from a peripheral hospital after a motorcycle accident six hours before admission. He had a tubular foreign body lodged above the suprasternal notch, with a history of torrential bleeding from the site of injury but that had since ceased. His relatives gave a positive history of momentary loss of consciousness followed by mental confusion immediately after the accident but no history of bleeding from the ear, nose and throat.

On primary survey, the airway was patent with regular quiet breathing and there was no cervical spine tenderness. The respiratory rate was 18 breaths per minute with an oxygen saturation of 97–99% on room air. The trachea was central with symmetrical chest expansion, and equal bronchovesicular breath sounds bilaterally. All his peripheries were warm and had a normal radial artery volume pulse with a capillary refill time of less than 3 s in all limbs. His radial pulse rate was 108 beats/minute and he had a blood pressure of 95/59 mmHg. His heart sounds S1 and S2 were heard and normal with no added sounds. The Glasgow Coma Scale was 10/15. His pupils were 3 mm in diameter bilaterally and reacting to light normally. There were no craniopathies or peripheral localizing signs. His random blood sugar was 5.8 mmol/L. There were bruises over the right frontal area but with no active bleeding. No other obvious deformities were evident. The eFAST (Extended Focused Assessment with Sonography in Trauma) was negative.

**Fig. 1:**
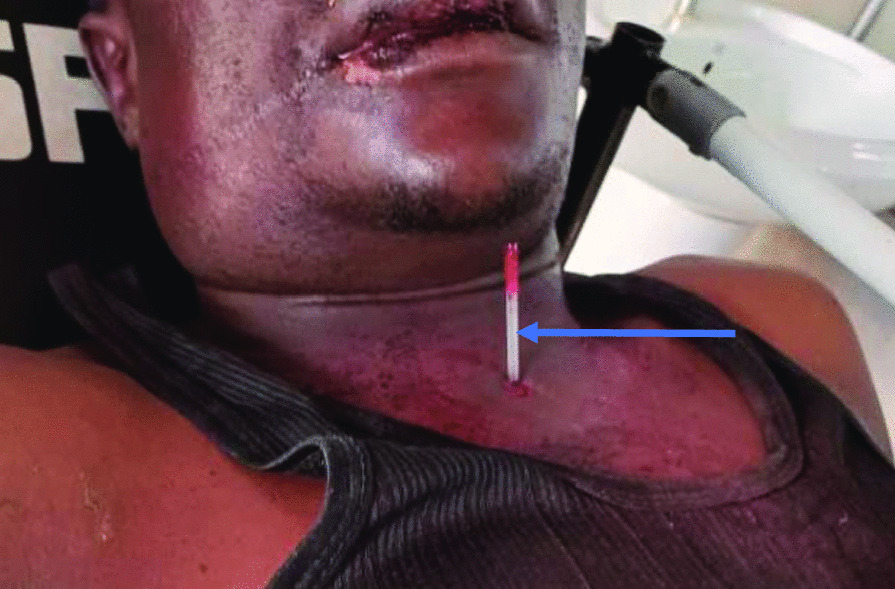
Preoperative photo showing the ball-point pen (indicated by the blue arrow) lodged above the supra-sternal notch

On secondary survey, he had an edematous right cheek and neck, multiple bruises on the right side of the mouth around the labial commissure, but no wounds visualized in the oral cavity. He had a normal ear, nose and throat examination. A ball-point pen plastic ink chamber (1.5 mm diameter) was firmly lodged in the midline about 1 cm above the suprasternal notch with no active bleeding. There was swelling in zone I and II of the neck and around the area of the point of entry by the pen (See Fig. 1). The rest of the secondary survey was unremarkable.

**Fig. 2:**
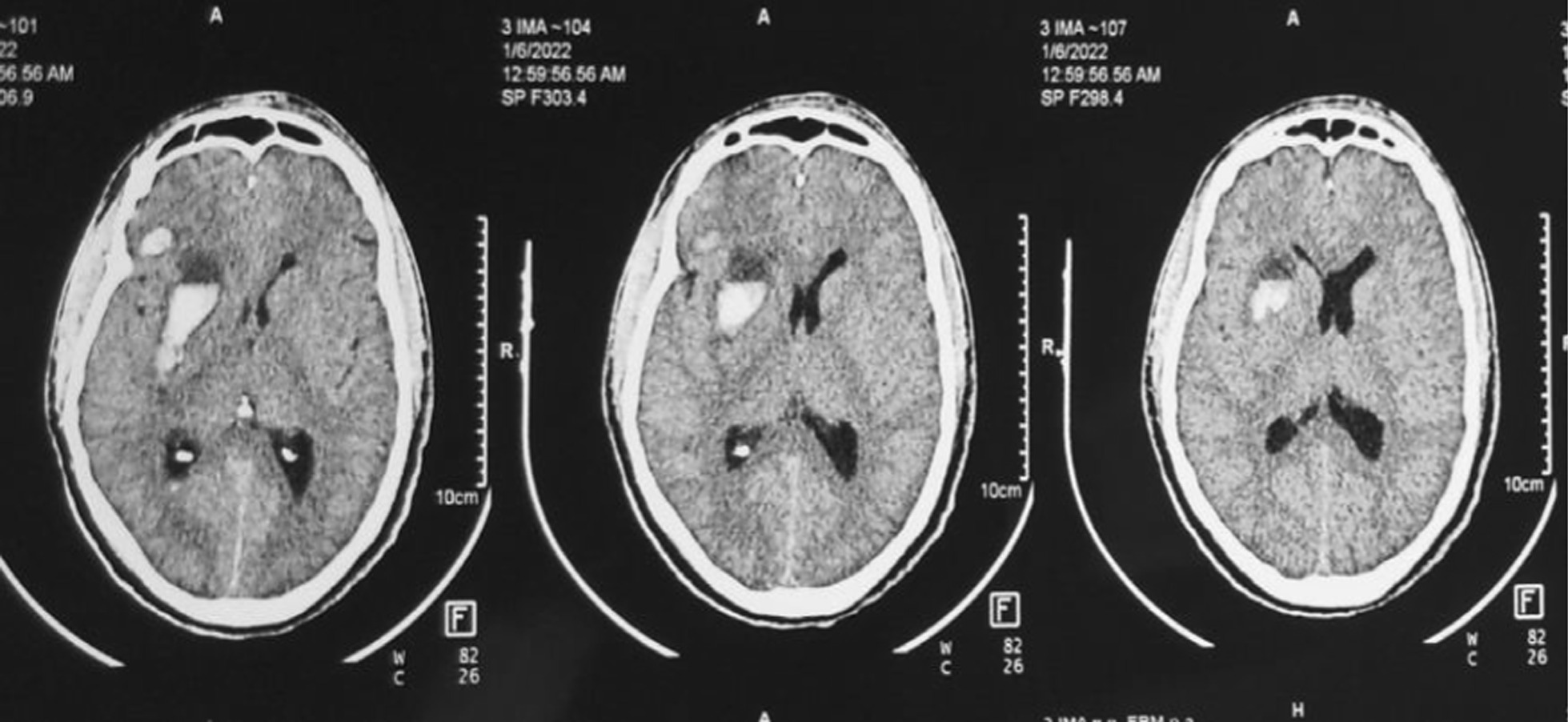
Deep right basal ganglia hemorrhage and right frontal contusions

**Fig. 3:**
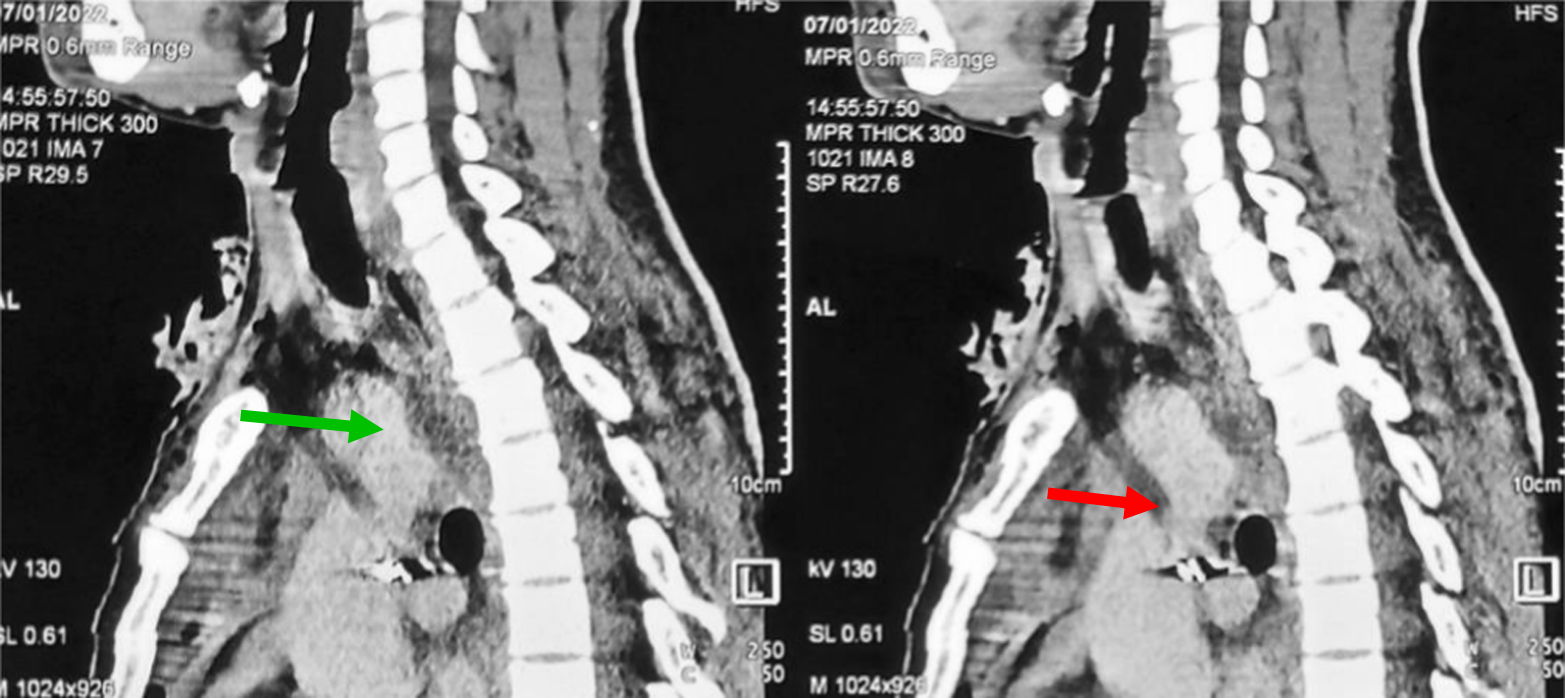
The green arrow is pointing to the brachiocephalic artery. The red arrow shows the track of the ball-point pen traversing through the superior mediastinum to penetrate the lumen of the aortic arch

He had a hemoglobin of 12.6 g/dL. The renal and liver function tests were all normal. The chest radiograph was normal. A non-contrasted brain CT-scan showed deep right basal ganglia and lateral ventricle haemorrhage with right frontal lobe contusion and intracerebral haemorrhage (See Fig. 2). The contrasted CT-angiogram of the chest demonstrated a penetrating foreign body traversing the superior mediastinum and penetrating the lumen of the ascending aorta just distal to the brachiocephalic artery origin but without perforating the posterior wall of the aorta (Fig. 3).

**Fig. 4:**
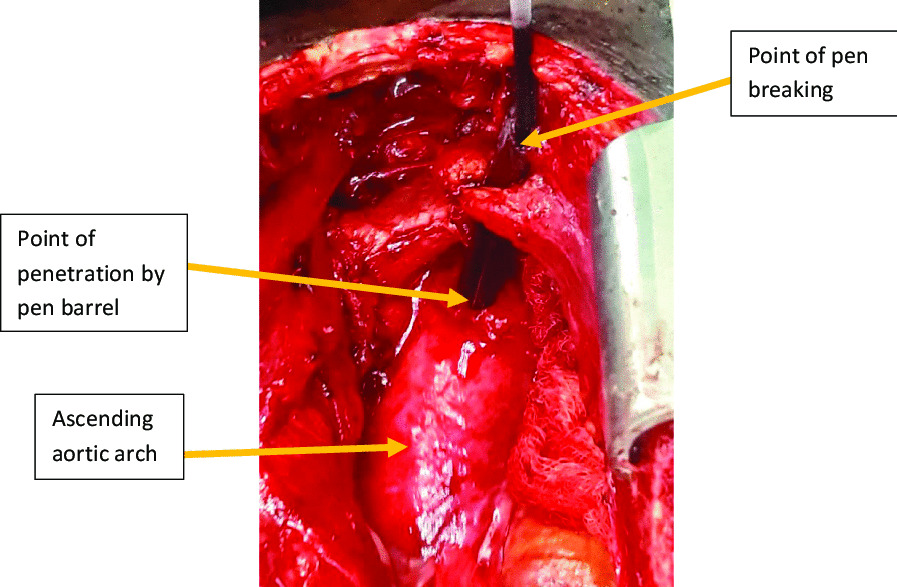
Ball-point pen penetrating the aortic arch

The patient underwent mediastinal exploration via a median sternotomy. We found a ball-point pen traversing the superior mediastinum and the anterior aortic arch wall (Zone 0) with the tip lying in the lumen of the aorta. Only the smaller plastic pen ink chamber was visible externally. The larger diameter and rigid pen barrel had broken within the mediastinum and was plugging the aortic perforation (Fig. 4). There was no obvious active hemorrhage or hematoma in the superior mediastinum and no injury was to the trachea, oesophagus or major aortic arch vessels.

**Fig.5:**
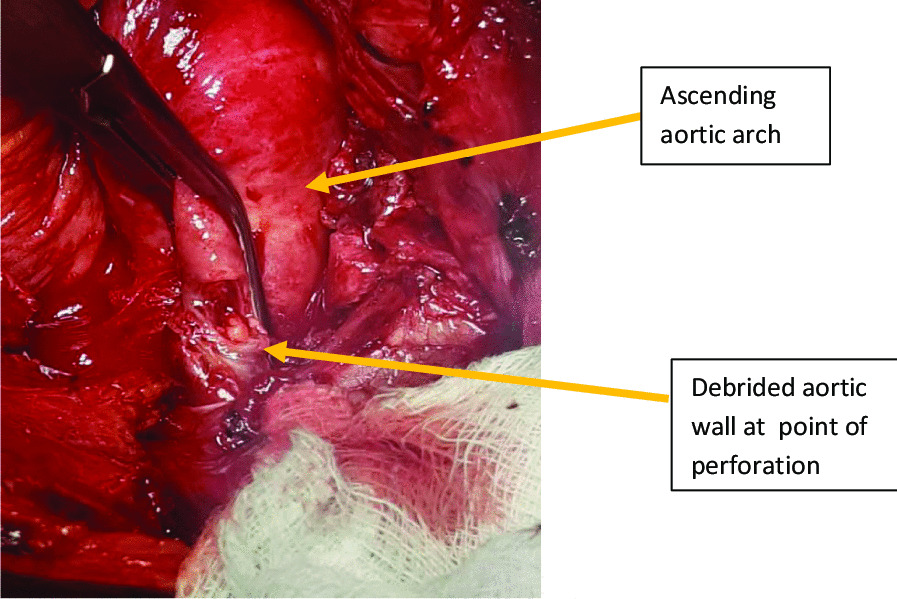
Defect on the anterior aspect of the aortic arch wall after removal of the ball pen indicated by the yellow arrow

**Fig.6:**
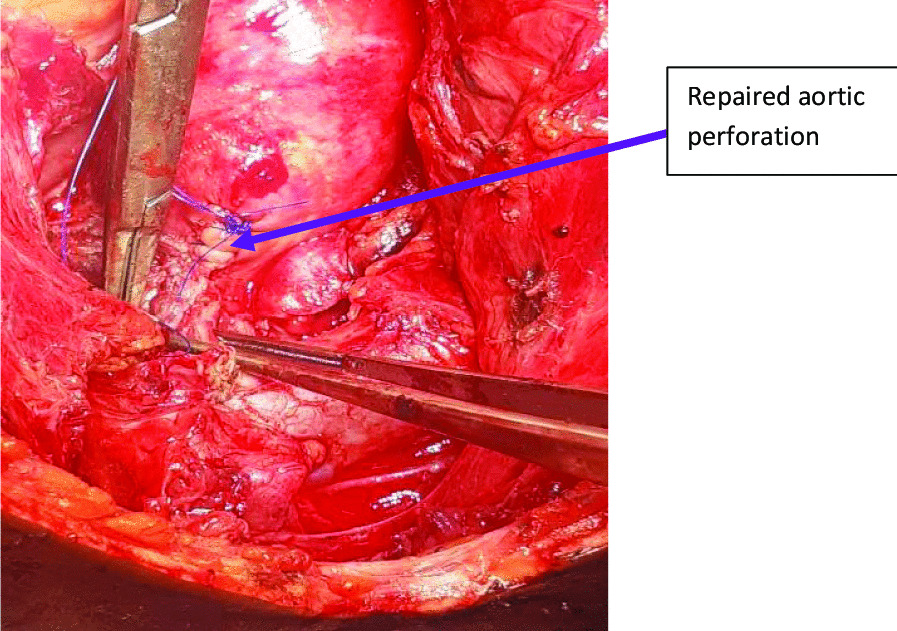
Repaired aortic arch wall defect indicated by the blue arrow

Dissection and mobilization around the aortic injury was done. Under systemic heparinisation, the ball-point pen was explanted and a side biting De Bakey aortic clamp was immediately applied to control haemorrhage (Fig. 5). A 10 mm full thickness injury in the anterior wall of the aortic arch was noted. The defect was debrided and repaired with a polypropylene 4/0 stitch in a double layer (Fig. 6). A 36-Fr mediastinal drain was left in situ. Hemostasis was achieved and the chest wall closed in layers.

The patient was transferred to the ICU for immediate postoperative management. He developed worsening confusion during the first two postoperative days which was treated with antipsychotics. The intracerebral haemorrhage was managed conservatively and no anticoagulation was given postoperatively. On the 4th postoperative day, he was transferred to the ward where recovery continued uneventfully but with residual mental confusion. On the sixth postoperative day the chest drain was removed and the patient was discharged home on antipsychotics, antibiotics and analgesics.

He was reviewed two weeks later and all wounds were noted to be healing well. However, he had residual mental confusion that was considered part of the post-concussion syndrome and for which continued neurosurgical review was sought. His review at 6 weeks revealed complete resolution of his mental confusion with no other neurological deficit and a healed surgical scar.

## Discussion

Injuries to the aortic arch are rare and carry a high incidence of morbidity and mortality[4]. Penetrating aortic arch injury from other mechanisms have been reported before including stabs from a knife and screw-driver injuries [2, 5]. An autopsy study done earlier reported pre-hospital death of all patients with penetrating injuries to the thoracic aorta and its arch branches while 5.5% of those with blunt injury were able to reach the hospital [6]. Therefore, this patient in our case report is one of the few cases with penetrating aortic injury to come to the hospital alive.

Our patient was preoperatively hemodynamically stable providing ample time to adequately investigate and prepare for mediastinal exploration. The chest radiograph appeared normal because of the absence of significant mediastinal bleeding that would provide a widened mediastinal shadow. The ball-point pen was predominantly plastic and hence radiolucent making the chest radiograph appear free of a foreign body. Contrasted CT angiography is considered the gold standard for investigating aortic and other thoracic vascular injuries in haemodynamically stable patients. It helps confirm the diagnosis by providing a detailed visualization of the aorta and adjacent structures [2, 5, 7]. It was essential in defining the location of the aortic perforation in this case and hence guiding in the surgical approach for explantation of the ball-point pen. In addition, chest CT helped in excluding other morbid states such as mediastinal haemorrhage, pneumothorax and pneumo-mediastinum as well as aortic dissection which could have required additional surgical interventions. Other helpful investigations that can be done for aortic perforations include trans-thoracic echocardiography (TTE) for lower ascending aortic injuries, and trans-esophageal echocardiography (TEE) which is recommended in descending thoracic aortic injuries [7]. TTE has its limitations in investigating aortic lesions especially of the descending aorta due to the poor echo windows resulting from limited access by the rib cage and inadequate clarity of the image due to air from the adjacent lungs [2]. However, because of its non-invasive nature and availability, TTE is helpful in quick evaluation of critically ill and unstable patients who require emergency intervention. In hemodynamically unstable patients, there is no time for carrying out investigations, therefore the team might proceed straight to an emergency operation based on the decision and assessment of the emergency team[8].

Postero-anterior and lateral chest radiographs are useful in imaging of aortic injuries and aid in defining the presence of pleural effusions that could suggest communication with the pleural cavity or inflammatory effusions induced by adjacent large haematomas. In addition, chest radiographs can show abnormal aortic shadows including widening of its outline suggestive of aortic dissection or major branch vessel injury with massive mediastinal haemorrhage [2, 4]. In this case, the chest radiograph appeared normal because of the absence of any haematoma. 

Most cases of aortic arch injury undergo prior medical stabilization followed either by either open surgical repair or endovascular repair [4]. Conservative management following a stab injury of the aorta has been reported in a case that remained haemodynamically stable throughout the hospital stay. Antihypertensive therapy and close monitoring in the hospital that are even continued at discharge have been recommended [4, 9]. Observation of limited strenuous activity as a lifestyle change is necessary to aid in blood pressure control however, there remains a risk of future pseudoaneurysm formation at the site of injury [9].

Approach to repair of aortic and arch vessel injuries is dependent on the site of injury. Ascending and arch vessels are best accessed via a median sternotomy. Other approaches include anterior thoracotomy for transverse and aortic arch branch artery injuries [4, 9]. The patient we present had an exploration via a median sternotomy. This was appropriate for the ascending aortic injury. However, it was also convenient for maneuvering and explanting the broken barrel of the ball-point pen.

The absence of a haematoma in the mediastinum was due to the plugging effect of the funnel shaped tip of the ball-point barrel and this helped in limiting haemorrhage. We repaired the aorta primarily with a running 4/0 polypropylene stitch as has been reported before. Larger, lacerations may call for patch repair or use of interposition grafts on cardiopulmonary bypass with or without deep hypothermic circulatory arrest [9, 10].

## Conclusion

Penetrating ascending aortic injuries require a high index of suspicion for early intervention and good outcomes. Haemodynamically stable patients should be fully investigated and the location of injury well elucidated to aid appropriate intervention. In this patient, the ball-point pen barrel acted as a seal at the site of injury preventing catastrophic exsanguination. Open surgical management via sternotomy provided opportunity for continued control of mediastinal haemorrhage.

## Data Availability

Data sharing does not apply to this article as no datasets were generated or analyzed during the current study.

## Abbreviations

CT: Computed tomography
ICU: Intensive Care Unit
OPD: Out Patient’s Department
